# Spirit-Lamp Explosions

**Published:** 1856-10

**Authors:** 


### SPIRIT LAMP EXPLOSIONS.

Singular and erroneous ideas in regard to the explosions of spirit lamps seem to be quite prevalent, both in and out of the profession. This remark is suggested by a notice, in the July number of the "News Letter," of a serious accident occurring to a distinguished member of the profession in New Orleans. We are surprised that so few explosions occur, when we consider the structure of many of the lamps in use. That style, with various slight modifications, called by Piggott, "the common form of the Dentist's lamp," and rep resented on page 241 of his "Dental Chemistry," is extensively used in some parts of the country. Now, why should it not explode? for, like the Irishman's Niagara, "it has nothing to hinder it," as will be seen, at a glance, by all who understand the cause of the explosions.

After noticing the accident, one of the editors of the "News Letter," like ourselves, feeling "inclined to say a word," winds up as follows:

"Lamps should have a spout of good length, should be kept, when using, well filled, and the wick should fit tightly, not so tightly, however, as to prevent the necessary absorption of the fluid—and should extend the whole length of the spout, at least. A vent in the top of the lamp is also desirable."

We like all these suggestions very much, *except the "vent."*

Before proceeding further, allow us to state that alcohol, whether in liquid or vapor, is not explosive. The lamp may become heated, or the fluid in it take fire, and it will boil over, perhaps with sufficient force to throw the lid off the "ordinary lamp;" if not, the liquid will run out at the spout, and, of course, it will burn. This, however, is not explosion; yet it is all that can be done toward it with alcohol, either in liquid or vapor, in an ordinary lamp.

Now, some may be as much startled at this as if we had presumed to answer that eternally open question, "Will saltpetre explode?" Well, when saltpetre is properly combined, in due proportions, with charcoal and sulphur, the question is no longer *open*, but the mass, assisted by a spark, is able to open almost any thing which attempts its confinement. So, while neither alcoholic vapor nor atmospheric air is explosive, a mixture of the two, within certain proportions, is highly so.

Here, then, lies the whole secret. The vapor is combustible, but, for want of a supporter, it can, ordinarily, burn only at the surface. But, when mixed with air, each atom of the combustible is in contact with a supporter of combustion, and the whole mass is instantly ignited, and an explosion is the consequeuce. Now, it is readily inferred that any form of lamp, even the most defective, may be used many times without an explosion, simply because the necessary mixture of air and vapor does not often take place. But, when the mixture, in proper proportions, is present, the flame will reach it, in spite of *tight wick or long spout*, as these terms are usually understood. The class of 1853 and 1854 in the "Ohio College" will remember a slight (?) explosion in the lecture-room, when the flame, to reach the reservoir, penetrated a blowpipe fifteen inches in length, with an unusually small orifice.

If the lamp becomes heated. in using, the space above the fluid is filled with vapor. As it cools, this is condensed and a partial vacuum occurs. The equilibrium is restored by an influx of air, unless, by the structure of the instrument, this is prevented. Now, if the mixture be in explosive proportions, the explosion takes place when the lamp is again lighted. Sometimes, the proportion of vapor is too small, and the accident does not occur till, by heating the lamp, its quantity is increased. The influx of air, however, is usually too slight to form an explosive mixture, and on this principle alone can the exemption from accident be explained.

The precautions we usually recommend are, to construct the lamp so that the body of it is not liable to be heated to any extent by use, so that the entire diameter of the spout will be filled as long as any fluid remains in the lamp, and so that the admission of air above the fluid can be totally prevented. The "vent in the top of the lamp" we think decidedly increases the danger. W.

